# Atraumatic Fractures in Multi-Morbid Older Adults: A Series of Five Cases and Review of Literature

**DOI:** 10.7759/cureus.51333

**Published:** 2023-12-30

**Authors:** Shaimaa N Rohaiem, Basim F Khan, Ghadeer H Al-Julaih, Ahmed S Mohammedin

**Affiliations:** 1 Geriatrics and Gerontology, Ain Shams University, Cairo, EGY; 2 Pediatrics and Child Health, King Fahd Hospital of the University, Al-Khobar, SAU; 3 Family Medicine, Eastern Health Cluster, Ministry of Health, Al-Khobar, SAU; 4 Internal Medicine/Geriatrics, King Fahd Hospital of the University, Imam Abdulrahman bin Faisal University, Al-Khobar, SAU

**Keywords:** multi-morbidity, osteogenic fever, frailty syndrome, osteoporosis, metabolic bone diseases, elder abuse and neglect, transfer and transport fractures, osteotoxic burden, spontaneous fractures, atraumatic fractures

## Abstract

Atraumatic fractures (ATFs) are a fragility fracture subtype with occasional medicolegal issues. ATFs are defined as fractures because of a “low-energy mechanism that is usually considered incapable of producing a fracture.” They are an underreported disorder, with epidemiological variations. ATF phenomena were previously reported not only in older adults, but also in children, young adults, older adults, and animals.

This study is a short retrospective case series exploring atraumatic fractures in a tertiary care university hospital. Over a period of two years, a total of seven ATF cases were identified. However, only five fulfilled the inclusion criteria. Local causes of pathologic fractures (e.g., metastasis) and elder abuse or neglect were excluded. Comparison of the cases’ clinical profile, fracture profile, and management was done.

All five cases were frail females with significant osteotoxic burdens from medications and multi-morbidities. ATF presentations included typical (as pain) and atypical (as painless, loud crack, and sudden giveaway) symptomatology. One ATF had a coincident unexplained aseptic fever. Three cases had more than one fracture (fracture cascade), confirmed and followed up by x-rays. All the cases were managed conservatively except for one case that underwent hip hemiarthroplasty. Plans of care included managing the osteotoxic multi-morbidities burden, focusing on the whole body, not only on the fracture or bone. The study provided insights about challenges in presentations of ATF (as the bone fracture acute phase reaction: osteogenic aseptic fever). Risk factors are classically assumed to be osteoporosis, but it is usually systemic and multifactorial. A high risk of fracture warning sign could help decrease ATF occurrence or fracture cascades. Four ATF categories were detected to help healthcare systems identify high-risk patients and raise awareness among medical staff, families, and caregivers. Future studies of the at-risk groups are needed to understand ATF knowledge gaps, challenges, and the best treatments.

## Introduction

Atraumatic fractures (ATFs) are a challenging subgroup of fragility fractures (reflecting advanced bone failure). They commonly occur in people with multi-morbidity, frailty, and/or hypokinesia (slow or diminished movement of body musculature) [1]. ATFs are defined as fractures because of a “low-energy mechanism that is usually considered incapable of producing a fracture,” excluding local diseases such as malignancy and cysts (known as pathologic fractures) [2,3]. These fractures may occur during normal or routine daily activities such as “lifting, moving, turning chairs and beds, or transferring to and from toilets” [4]. American researchers stated that “Long bone fracture in the absence of trauma in patients after prolonged bed rest is an important geriatrics phenomenon to be recognized” [5].

Multiple terms were assigned to this “phenomenon” of extreme fragility depending on different points of view (e.g., mechanical, triggers, pathology). While some authors used trigger-related terms, e.g., transfer fractures, others used fracture-mechanics terms (e.g., fatigue fracture) [6,7]. Other professionals stated that “the following terms are synonymous with pathological fracture: insufficiency fracture, spontaneous fracture, non-traumatic fracture, and non-traumatic compression fracture” [8]. Another British team used a “combined category” while discussing the causes of fragility fractures, stating it as “spontaneous, pathological, stress or insufficiency fractures” while studying older adults' fractures [9].

An American researcher has used an ATF “combined category,” commenting that “it has been almost three decades since these types of fractures, referred to as minimal trauma fractures, spontaneous fractures, spontaneous long bone insufficiency fractures, and transfer and turning fractures, were first described in nursing home residents” [6]. Readers can easily notice terms “interchangeability” in the same article [5,7,10-12]. This reflects the absence of a single fully descriptive term expressing full ATF aspects. To facilitate discussion throughout this article, we are using the unified term “atraumatic fractures.”

ATF is a not uncommon phenomenon (maybe underreported) with epidemiological variations [13,14], with “most cases are reported as case series, with only a few epidemiological studies” [15]. Studies showed an incidence range from 1% to 15% [16]. An Italian predictive model estimated ATF incidence as 5-10% of all fractures [17]. Studies estimated ATF incidence as follows: (a) 0.84 per 100 subjects per year (American nursing homes) [4], (b) 1.3% in oldest old residents (French nursing homes) [18], (c) 0.8% in British older adults presenting with a fracture as a “sum-up category” [9], and (d) 3-4.4% (Japanese hip ATF study) [19]. A cross-sectional New Zealander study found that 11 out of 815 hip fractures “were reported as occurring spontaneously, i.e., before falling” [20]. The ATF’s epidemiologic differences are explained by differences in the study populations, settings, methodology, and the studied bone(s).

It is not uncommon for ATF to cause “medicolegal dilemmas,” avoidable suffering, or anxiety [5]. An ATF has an unexplained/vague history, “is often met with suspicion of abuse or mistreatment” and “may be mistaken as patient abuse by the staff [4,21]. An American report pointed out that “mistakenly characterizing a spontaneous bruise or other injury (as fractures) as intentionally inflicted (abusive) may lead to substantial clinical, social, and legal jeopardy for all concerned” [22]. Past reported cases included families suspecting iatrogenic abusive fractures (at hospitals or nursing homes) [5,18,23]. Home care-related ATFs (presenting to hospitals) were suspected of elder abuse or neglect (EAN) by healthcare providers [11,13,24]. Investigating these cases has excluded EAN by history and examination and could not find EAN evidence. This requires good experience and skills to avoid overdiagnosis/underdiagnosis of EAN [11].

American researchers concluded that ATF occurred because of (1) mild trauma during the provision of usual care (witnessed or unwitnessed) or (2) without significant trauma (as reported by cognitively intact patients) [4]. An Irish study has pointed out that ATF “should be regarded as a symptom of an underlying predisposing or precipitating condition” [11]. The exact etiology of ATF is vague as many geriatric syndromes. It has a systemic multifactorial osteotoxic burden (OB) rather than localized [11,17,25]. Disorders affecting bone metabolism are known as metabolic bone diseases (MBDs). MBDs was a common classic term before being replaced by the histopathologic and radiologic term “osteoporosis.” Exclusive focusing on osteoporosis leads to ignoring other MBDs and ends up treatment solely with “antiosteoporotic medications.” Many cases that are unfit for these medications are left without any care plan. Many of the MBDs’ OB is reversible or modifiable but undertreated due to focus on the antiosteoporotic medications. Revisiting the Utah paradigm of skeletal physiology (combining anatomical, clinical, pathological, and basic science) can help identify disorders contributing to ATF [26,27].

American researchers have discussed theories of bone physiology, to answer “why some patients with osteoporosis develop spontaneous fractures but others do not?” [27]. They commented that spontaneous fractures are not spontaneous but related to bone strain and microdamage. They discussed “classifying osteopenias and osteoporosis” in a new manner. Then they discussed the possibility of spontaneous fracture in four kinds of osteoporosis with similar bone mass and Z-scores. For example, the last type (caused by chronic disuse and muscle weakness from systemic disorders) is labeled as transient osteopenia and naturally reversible osteopenia [27].

Proper OB management requires getting out of the “osteoporosis term frame,” into the term of metabolic bone disorder (or osteodystrophy). Two similar updated medical terminologies, emphysema (now chronic obstructive pulmonary disease) and diastolic dysfunction (now heart failure with preserved ejection fraction), were revised. Instead of previous broad troublesome usage, they were restricted to clinical practice into limited use in radiology and histopathology. We need to manage osteotoxic burden as a multifactorial geriatric syndrome. Proper understanding of ATF contributes toward preventive management of any modifiable osteotoxic burden and minimizing medico-legal dilemmas. Given this need, the present study attempted to understand osteotoxic burden and challenges of ATF management.

This is a short case series of older adults (>60 years old) diagnosed with atraumatic fractures (ATF) at King Fahd Hospital of the University in Alkhobar city (a 400-bed tertiary care hospital). ATFs were defined as “a fracture caused by a relatively low-energy mechanism that normally would not be expected to cause a fracture” [2,3]. Exclusion criteria were cases with pathologic fractures due to local bone disorder (malignancy or bone cyst), cases with high-energy trauma, or confirmed abusive fractures.

One case from this case series was previously presented as a poster at the 29th Annual Internal Medicine meeting on December 13, 2018, in Al-Khobar, Saudi Arabia.

## Case presentation

Background of cases

Data on the cases were collected from medical records from general medical units or geriatrics division from January 2017 to December 2019. No cases were detected under the services of orthopedics, endocrinology, rheumatology, or neurology. Medical records were reviewed by two independent doctors to extract data. Missing data were collected by contacting the patients, family caregivers, or attending healthcare providers whenever possible. The collected data included patient demographics, comorbidities, fracture event analysis (e.g., symptoms and signs of fracture), investigations, management (e.g., orthopedics surgery versus conservative management), complications during hospitalization (e.g., fracture cascade), and outcome whenever possible. All five cases were older adult females with significant osteotoxic burden and multiple comorbidities, such as hypokinesia (5/5), severe frailty (4/5), diabetes mellitus (5/5), severe contractures (1/5), cerebrovascular strokes (3/5), dementia (2/5), chronic kidney disease (2/5), osteoporosis (3/5), primary hyperparathyroidism (Case 4), adrenal insufficiency (Case 5), and nasogastric tube feeding (3/5 cases). All cases chronically used osteotoxic medications (such as proton pump inhibitors, furosemide, and antiepileptics). All were diagnosed with ATF based on history, examination, radiology, and lab investigations.

Case 1

The patient was an octogenarian female with multi-morbidity (Table 1). She was bedridden (due to vascular dementia) and dependent on her activities of daily living (ADLs) over the past six years. She had a nasogastric tube and a tracheostomy (after prolonged mechanical ventilation for pneumonia). She had recurrent admissions with urinary tract infection (UTI) and dehydration. She was admitted with another UTI and then had obstructed discharge due to family caregiving issues. Trials of transfer to long-term care and/or home with home healthcare follow-up were unfruitful for more than one year. During her hospital stays, she was accompanied by a formal caregiver provided by the family. One day, the daughter noticed a swelling at the left lower arm and elbow of her mother. There was no history of trauma, falls, or convulsions, apart from doing the usual routine daily nursing care of the bedridden people. She was regularly on aspirin, haloperidol, phenytoin, vitamin D3, and pantoprazole. On examination, the patient was vitally stable, the arm was swollen with deformity, tenderness, and facial grimacing, but there was no ecchymosis. Otherwise, the examination was unremarkable.

**Table 1:** Summary of clinical data of the five atraumatic fracture cases. AF: atrial fibrillation; CKD: chronic kidney disease; DEXA: dual x-ray absorptiometry; DM: diabetes mellitus; HF: heart failure; HTN: hypertension; IHD: ischemic heart disease; N/A: not available; NGT: nasogastric tube; OP: osteoporosis; PPI: proton pump inhibitors

Characteristics	Case 1	Case 2	Case 3	Case 4	Case 5
Frailty and functional status	Severely frail, non-ambulant (bedridden) from multiple old strokes	Severely frail, non-ambulant (wheelchair bound) from an old stroke	Severely frail, non-ambulant (bedridden) from multiple old strokes	Mildly frail limited ambulation (homebound) from knee osteoarthritis and Charcot’s neuroarthropathy	Moderate to severely frail, very limited ambulation (wheelchair bound) from frailty
Multi-morbidity and risk factors	Frailty, DM, HTN, strokes, vascular dementia, epilepsy, OP, on NGT feeding, and tracheostomy	Frailty, DM, stroke, vascular dementia, OP, HF, and NGT feeding	Frailty, DM, AF, strokes, vascular dementia, multiple contractures, CKD, primary hyperparathyroidism, fragility fractures, epilepsy, neurogenic bladder on suprapubic catheter, and NGT feeding	Frailty, DM, IHD, HTN, CKD, OP, adrenal insufficiency, mild dementia, and Charcot’s neuroarthropathy	Frailty, DM, diabetic nephropathy, diabetic retinopathy, immobility, and chronic constipation
Osteotoxic drug	PPI, phenytoin, and haloperidol	PPI and furosemide	PPI, phenytoin, and phenobarbital	PPI and furosemide	Furosemide
Presentation	Daughter noticed - new swelling and deformity	Caregiver noticed - painful swelling and bluish patch on the left thigh and unexplained fever	1st fracture: caregiver noticed - painful swelling of left lower leg, and fibula fracture noticed at follow-up; 2nd fracture: nursing noticed - loud crack sound while turning the patient in bed	Patient noticed - painless loud crack sound, sudden giveaway while standing, and inability to walk	1st fracture: patient noticed - right painful limitation of hip movement while standing from toilet; 2nd fracture: patient noticed - painful limitation of knee movement while standing from bed
Fracture site and description in x-ray	Left humerus: spiral fracture of the mid-shaft with angulation and displacement	Left femur: displaced comminuted distal metaphyseal fracture, compression fracture of 2nd thoracic vertebral body	1st fracture: displaced transverse fracture of the left lower tibia; 2nd fracture: displaced spiral fracture of the distal femoral shaft	Right femur: displaced spiral comminuted fracture	1st fracture: displaced fracture of the right femoral neck; 2nd fracture: displaced comminuted fracture of the distal right femur
DEXA scan T score	-2.8 lumbar spine (five years pre-fracture)	N/A	N/A	-2.7 spine -3.2 left femur	N/A
Fracture outcome	Immobilization by a cast achieved complete healing with malunion	Immobilization with cast achieved full healing	Immobilization of the left leg by back slab (cast postponed due to swelling), healing was achieved. Then she sustained another 2nd ATF and died due to sepsis	Immobilization with skin traction, but discharged against medical advice. Then lost follow-up	1st fracture: uneventful right hip hemiarthroplasty; 2nd fracture: immobilization with above knee cast

**Figure 1:**
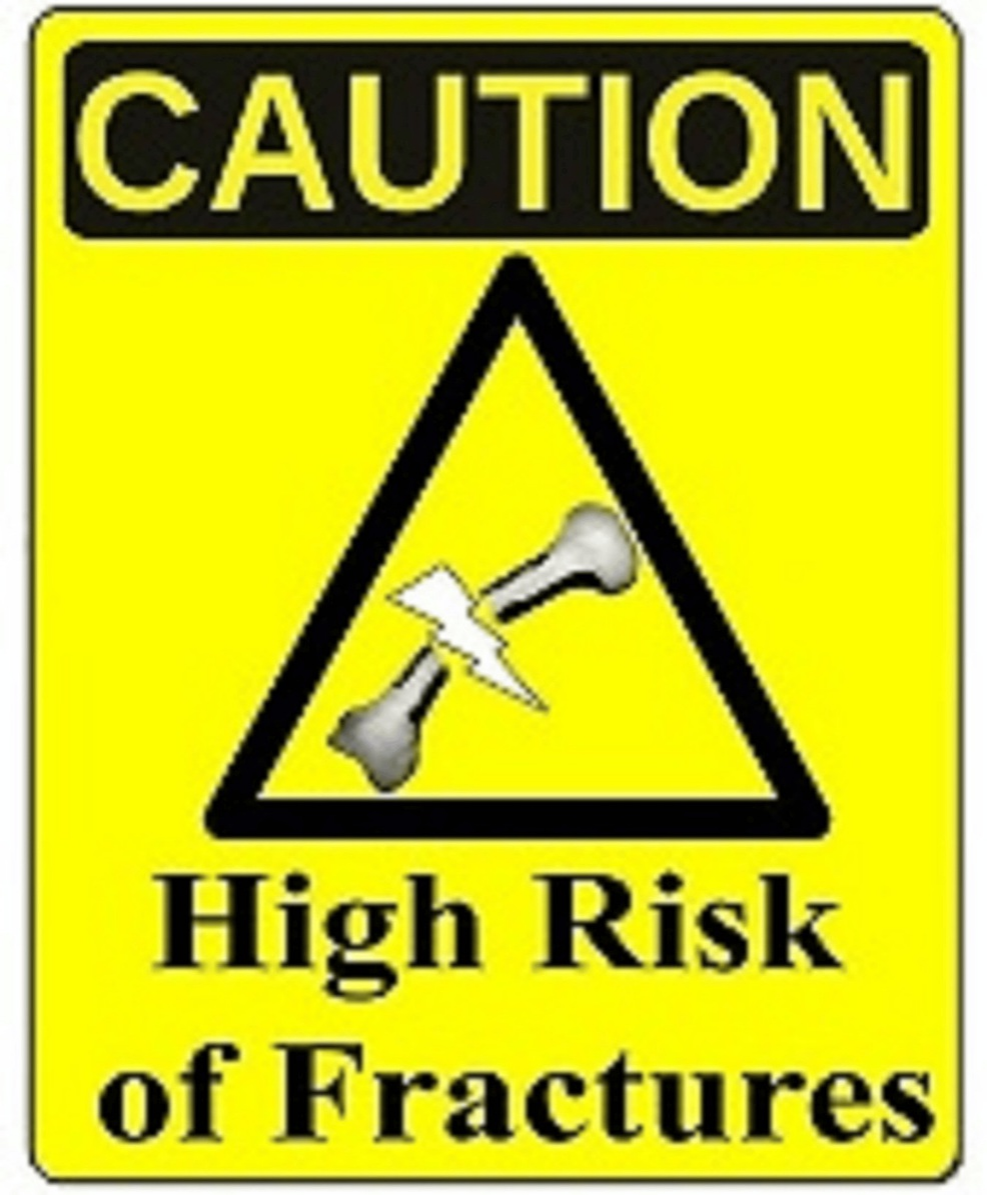
A warning sign for high risk of atraumatic fractures. The yellow triangle symbolizes caution. The lightning signifies an unexpected dramatic event, possibly painful, sometimes with shocking elements, such as loud crack or acute phase reaction.

As shown in Table 2, lab work-up was within normal (no new vitamin D levels were available). X-ray confirmed the presence of spiral fracture at mid-shaft of left humerus with angulation and displacement, and generalized reduction in bone density (Figure 1). There was no evidence suggestive of malignancy or bone cysts by radiology or lab. The daughter filed a complaint against healthcare providers claiming elder abuse or neglect. After formal investigation, the fracture was labeled as an atraumatic fracture (ATF). After diagnosis of the fracture, the daughter recalled a positive history of an old (>five years back) ATF at the base of the right little finger, with no identifiable trauma, and was treated conservatively. The patient was diagnosed, such as ATF of the left humerus during hospital routine daily care by healthcare providers or her formal caregiver. After immobilization by a cast, the patient achieved complete healing with malunion in one-month time. Other general care measures were applied as mentioned in this study and Table 1.

**Table 2:** Laboratory work-ups at the time of fractures. *Five years earlier before the fracture the value. **Four years earlier before the fracture the value was 13 pmol\L. Lab sum up - calcium levels were within normal range in all cases, phosphorus was high in Case 3, alkaline phosphatase was high in most cases, parathyroid hormone was triple times high in Case 3, and albumin was low in all cases. Vitamin D level was low in 4/5 of the cases at the time of fracture with secondary hyperparathyroidism.

Blood lab item (normal range)	Case 1	Case 2	Case 3	Case 4	Case 5
White blood cells (4-11 k/µL)	7.4	11	10	7.5	4.7
Hemoglobin (12-16 g/dL)	9.5	10.5	10.2	12	12.7
Platelets (140-450 k/µL)	318	301	334	231	140
Blood urea nitrogen (7-18 mg/dL)	17	30	42	16	22
Creatinine (0.6-1 mg/dL)	0.34	1.2	2.2	1.1	0.6
Sodium (135-145 mEq/L)	130	145	137	139	137
Potassium (3.5-5.1 mEq/L)	4.8	4.5	4.3	4.1	4
Total protein (6.0-8.0 g/dL)	7.3	7.5	7	7.1	7
Albumin (3.5-4.8 g/dL)	3	2.5	2.3	3.3	1.8
Alanine transferase (15-37 U/L)	28	40	19	27	23
Aspartate transferase (14-63 U/L)	36	20	14	44	25
Direct bilirubin (0.05-0.2 mg/dL)	0.2	0.2	0.1	0.1	0.2
Total bilirubin (0.2-1.2 mg/dL)	0.6	1.2	0.2	0.8	0.8
Alkaline phosphatase (46-116 U/L)	181	242	239	92	852
Gamma-glutamyl transferase (5-55 U/L)	60	22	24	27	55
Lactate dehydrogenase (81-234 U/L)	180	333	136	198	281
Calcium (8.5-10.1 mg/dL)	8.7	8.2	9.9	8.1	8.9
Phosphorus (2.6-4.7 mg/dL)	4	3.3	5	2.8	3.4
Vitamin D level (25-80 ng/mL)	13.7*	26.3	5.8	5.9	6.3
Parathormone (1.58-7.2 pmol/L)	3.09	15.9	110**	9.3	2.56
HbA1c (4-6%)	5.5	6	13	9.2	4.5
Prothrombin time (12.9-15.9 s)	12	13	12.2	14.2	14
Partial thromboplastin time (24.1-34.7 s)	29.4	38.3	28.4	24.3	35.7

Case 2

The patient was an octogenarian female with multi-morbidity requiring assistance in ADLs for >one year then she became dependent over the last two months before admission (due to strokes) (Table 1). Admitted with pneumonia, malnutrition, and deconditioning syndrome. She was improved and then discharged home. After nine days from discharge, she presented to the emergency department with unexplained new onset of fever (38°C) and large bluish patch (ecchymosis) in the left lower thigh overlying a tender swelling noticed in less than one-day duration. It was noticed by her daughter just after giving her a shower at home. Emergency physicians were suspicious of the unexplained history and the atypical presentation. There was no history of trauma, falls, or convulsions, apart from the recent bathing at her home bathroom and doing the usual routine daily nursing care. She was regularly on bisoprolol, atorvastatin, vitamin D3, calcium carbonate, dabigatran, furosemide, and pantoprazole. On examination, the patient was vitally stable, there was marked swelling in the left thigh with a blue ecchymosis, otherwise, the examination was unremarkable. As shown in Table 2, lab work-up was within normal limits (except for low normal vitamin D). Venous thromboembolic disorder was initially suspected, then excluded by normal venous duplex and CT pulmonary angiography. An incidental finding of compression fracture at third thoracic vertebral body was noted on a background of osteoporotic bone. An x-ray of left thigh was requested. It showed a displaced comminuted distal metaphyseal fracture of femur and a generalized reduction in bone density. No evidence was suggestive of malignancy or bone cysts by radiology or lab. Patient was diagnosed with ATF of the left femur during routine home daily care. After immobilization with cast, the patient achieved full healing in seven months. Other general care measures were applied as mentioned in the article and Table 1.

Case 3

The patient was an octogenarian female with multi-morbidity, bedridden for >three years (due to advanced vascular dementia), neurogenic bladder (on permanent suprapubic catheter), and recurrently admitted (four times over the past year) with catheter-associated urinary tract infection (CAUTI) and dehydration. She was regularly on phenytoin, phenobarbital, lispro insulin, and pantoprazole. She was admitted with urosepsis and acute kidney injury secondary to CAUTI. While admitted, the daughter noticed a painful swelling in her lower left leg. She claimed that it occurred due to a healthcare provider examination one day earlier. The daughter filed a complaint claiming elder abuse or neglect. After a formal investigation, the fracture was labeled as an ATF. On examination, the patient was vitally stable. The examination was normal apart from bilateral knees and ankle contractures, tender swelling, and edema of the left lower leg. The overlying skin was warm but without discolorations. As shown in Table 2, lab work-up was within normal limits (except for low normal vitamin D). X-ray showed a displaced transverse fracture of the left lower tibia and a generalized reduction in bone density. There was no evidence suggestive of malignancy or bone cysts by radiology or lab. Then the daughter recalled (retrogradely) that her mother was diagnosed with primary hyperparathyroidism (a parathyroid adenoma five years ago in another hospital). At that time surgery was offered but the family refused and did not follow-up. She also recalled an old ATF of her mother’s right foot treated with a splint (about three years back in another hospital) and an ATF of right tibia and fibula eight years back treated with a cast. Patient was diagnosed with ATF of the left femur and tibia while providing routine hospital daily care, secondary to hyperparathyroidism. Endocrine consultation was advised for surgery, but the family refused further investigation or surgery. Immobilization by a below-knee back slab was applied, cast was postponed by orthopedics as there was significant swelling and leg edema. Other general care measures were applied as mentioned in the article. Unfortunately, four weeks later the nurse heard a loud crack while repositioning and turning her in bed for diaper change and bathing. An x-ray confirmed the presence of a new spiral displaced fracture involving the right distal femoral shaft. Patient was diagnosed with ATF in right femur. Unfortunately, her condition rapidly deteriorated due to pneumonia and urosepsis. Then she died due to septicemia.

Case 4

The patient was a septuagenarian female with multi-morbidity (Table 1). She has been homebound with limited mobility for the past 15 years (due to severe knee osteoarthritis). While trying to stand from a chair, she had a sudden giveaway, inability to walk, and a painless, loud crack sound. She was brought to the emergency department by her family. Emergency physicians were suspicious of an unexplained fracture from the discrepancy in the family witnesses’ stories regarding the event and the patient denied having pain several times. She was regularly on bisoprolol, atorvastatin, glargine insulin, hydrocortisone, aspirin, furosemide, and pantoprazole. On examination, the patient was vitally stable, there was limitation of movement in the right hip, with internal rotation, but no skin bruises. She had old, deformed Charcot’s joints at the right knee and ankle, otherwise, the examination was unremarkable. As shown in Table 2, lab work-up was within normal limits (except for low normal vitamin D). X-ray was done and showed a displaced spiral comminuted fracture of the right shaft of the femur with multiple bony fragments, shortening, destruction of the right knee joint with bony fragmentation and debris (Charcot’s joint), and generalized reduction in bone density. The patient was diagnosed with a right shaft of the femur ATF during home routine daily care. Orthopedics managed the fracture by skin traction. The patient’s family requested discharge against medical advice after a few days (due to social reasons). A follow-up x-ray four months later showed interval angulation and further displacement of the right femur fracture, with extensive callus formation and soft tissue calcification. Then, unfortunately, the patient lost to follow-up.

Case 5

The patient was a hexagenerian female with multi-morbidity who needed assistance in both activities of daily living and instrumental activities of daily living due to frailty (Table 1). The patient felt pain in her right hip while getting up from a toilet seat and faced difficulty in walking. The pain was dull aching moderate in severity (6/10 in grade), aggravated by movement, and relieved by rest. The pain progressed and eventually prevented her from walking. She was brought to the emergency room with pain. She was regularly on glargine insulin, lactulose, furosemide, and spironolactone. On examination, the patient was vitally stable, there was painful limited movement in the right hip, but no skin bruises. There was bilateral pitting pedal edema. Otherwise, the examination was unremarkable. As shown in Table 2, lab work-up was within normal limits (except for low normal vitamin D). X-ray showed a displaced impacted fracture of the right femoral neck and generalized reduction in bone density. The patient was diagnosed with ATF of the right neck of the femur during home routine daily care. The patient was managed with a right hip hemiarthroplasty. She has restored most of her premorbid ADLs after rehabilitation. Other general care measures were applied as mentioned in the article and Table 1. However, two years later she had pain in the right knee while trying to stand from bed. X-ray showed a displaced comminuted fracture of the distal right femur. Orthopedics advised for above knee cast. Unfortunately, the patient lost to follow-up.

Characteristics and management of ATF cases

We identified seven ATF cases (two males and five females). The first male case charts had many missing data and we could not contact the caregivers. Caregivers of the second male case did not agree to be included in the study. Five female ATF cases fulfilled the inclusion criteria. We discussed the background of all cases above and summarized the case data in Table 1. ATF occurred during routine daily activities, usual nursing care, or accidentally discovered, and then treated by orthopedics. Medical care plan included managing the osteotoxic burden by deprescribing inappropriate osteotoxic medications and adding antiosteoporotic medications as needed, managing malnutrition, physiotherapy, and health education to families and healthcare providers by introducing warning signs for high-risk of fractures (Figure 1).

Fracture event profile

The mode of injury depended on mobility status. For active people, such as Cases 4 and 5, the mode of injury was while standing from a chair or toilet seat. Presentations of ATF showed variability as follows: (a) typical presentations (as swelling), and (b) atypical presentations (as loud crack, painless sudden giveaway, and unexplained aseptic fever. Regarding pain, Case 1 and Case 4 had painless fractures. Case 1 had advanced dementia and dysphasia, facial pain scale was negative with no behavioral or psychological changes. Specifically, Case 4 was fully conscious, homebound with limited mobility, mild dementia (but no communication issue), and Charcot’s neuroarthropathy in the right knee and ankle joints. She had a painless femur fracture (directly confirmed by the patient and the attending caregivers), even without other signs of pain, such as facial grimacing, agitation, or behavioral changes. Fracture cascades occurred in the third and fifth cases.

Lab and radiology profile

We reviewed specific bone-related labs and plain x-rays to guide the management plan (Table 2). Fracture morphology by x-ray was transverse (Cases 2 and 3), spiral (Cases 4 and 5), and angulated displaced fracture (Case 1).

Management and outcomes

There were no available antiosteoporotic medication records. Three cases had conservative orthopedics management by casting, slabs, or skin traction. Case 5 had hemiarthroplasty (as she was ambulant), then had above-knee casting for the second fracture. Two of the cases’ caregivers filed official complaints to the hospital administration claiming EAN. Official investigations concluded the fractures were ATF during the usual routine care. Family meetings were done with the families to explain the nature of ATF and the plan of care. Families accepted the diagnosis and were satisfied.

## Discussion

Considering bone quality, not just quantity

Osteoporosis is commonly assumed to cause fragility fractures. ATF risk factors are assumed to be primary or secondary osteoporosis. Literature has discussed the occurrence of ATF in the absence of osteoporosis [17]. Researchers studied ATF in permanent vegetative states (during daily care), stating that “fractures occurred independently of age or immobilization period and with no correlations to BMD at hip and spine or bone turnover markers” [28]. An Italian team stated that ATF pathogenesis cannot be attributed to the histopathological term “osteoporosis.” Additionally, we emphasize that it cannot be attributed to the radiological description of “decreased bone mineral density” (BMD) that is usually correlated with “bone quantity” [17]. Researchers have urged addressing bone quality to understand spontaneous fractures of the spine and bone failure [17,29].

Researchers from the Netherlands studied spontaneous vertebral fractures in (a) steroid-free patients with active Crohn's disease and (b) steroid-dependent patients with inactive or quiescent Crohn's disease [30]. Half of the patients with fractures had normal T-scores. They found that “the fracture rate was similar in both subgroups and was not correlated with the bone mineral density of the lumbar spine.” American researchers studied (by infrared analysis) collagen cross-linking in bone biopsies of spontaneous low-trauma fractures [31]. The study included three following groups: (1) high-turnover osteoporosis, (2) low-turnover osteoporosis, and (3) normal premenopausal women with normal BMD. Results showed that all three ATF groups had significant abnormal bone collagen quality despite having normal BMD in the third group.

Unfortunately, many textbooks, handbooks, articles, and guidelines of medicine have reduced the discussion and management of metabolic bone diseases and osteoporosis. Metabolic bone diseases "category term" is not used in contemporary textbooks, handbooks, articles, and guidelines except in prematurity/neonatology and nephrology specialties. Even respected professional societies in medicine, family medicine, endocrinology, orthogeriatrics, and orthopedics are not producing guidelines for managing metabolic bone diseases (which include lots of reversible osteotoxic burden, such as protein-energy malnutrition, hypokinesia, and frailty). Their current guidelines have been reduced to "osteoporosis management" (maybe even more reduced to postmenopausal osteoporosis). Sadly, some guidelines were more reduced to include only pharmacologic management of osteoporosis without any non-pharmacologic management (various reasons have caused this concept shift, one is ignoring this aspect in research). Perhaps older generations of physicians are aware of metabolic bone disease management, but lots of younger generations only provide a relatively narrow tube vision of pharmacologic management to osteoporosis for any metabolic bone disorders. No one can blame them as the histopathologic/radiologic osteoporosis term has nearly replaced the metabolic bone disease category. If one discusses metabolic bone disease with young generations, they find it a strange term and will not be able to read about it in medical textbooks, handbooks, articles, or practice guidelines. Younger physicians usually think it is an old abandoned term. We need to return to using "metabolic bone disease category" in our textbooks and guidelines, especially in multi-morbid people. Otherwise, lots of reversible osteotoxic burden would be ignored causing more ATF cascades. This does not mean abandoning the "histopathologic/radiologic osteoporosis" term, but it encourages detecting and managing the whole osteotoxic burden for expected better outcomes.

Osteotoxic burdens in multifactorial syndrome

Atraumatic fractures (ATF) are a challenging geriatrics phenomenon [5]. This phenomenon was also noticed in young spina bifida and cerebral palsy patients (with a false suspicion of child abuse and neglect) and described as spontaneous fracture syndrome in animals [5,32]. ATF occurred in several at-risk groups with advanced MBDs. ATF can be divided into four categories according to the osteotoxic burden (OB).

The first category is ATF in MBDs with predominantly malnutritional disorders. Malnutrition and MBDs were studied in human and animal studies. Malnutrition causes sarcopenia and frailty with a higher risk of fractures [11,33]. Weight loss is associated with bone loss, impaired bone macro- and microstructure, and increased fracture risk in older adults [34]. It affects calcium, phosphate, vitamin D deficiency, and other micronutrients, such as copper [35,36]. Human malnutrition-related ATF cases included severe protein-calorie malnutrition (PEM) [11,37,38], tube feeding-associated malnutrition (as in the cases of present study) [39], anorexia nervosa [40], and bariatric surgery malnutrition [41]. Human ATF was reported in the nutritional stressful states of gestation and lactation [42]. It is called “lactational osteoporosis” for gestation and/or lactation in animals’ ATF pathology [43]. According to an animal pathology textbook, “most cases of osteoporosis in animals, especially farm animals, are nutritional in origin and may be caused by a deficiency of a specific nutrient, such as calcium, phosphorus, or copper, starvation, restricted and/or imbalanced intake.” It also highlights multiple mechanisms of malnutrition-induced MBDs [44]. Nutritional animal ATF outbreaks were reported in cows, associated with PEM, calcium mobilization for lactation, and copper deficiency [45-47]. Collagen content was significantly higher in the humeri of cows without fractures, while total collagen crosslink content was significantly higher in the humerus of cows with spontaneous fractures, indicating that PEM might be more important than copper deficiency [48]. Outbreaks of spontaneous fractures due to nutritional osteoporosis with copper deficiency occur in pigs [49]. A case series of spontaneous fracture syndrome in dairy cows was associated with transient osteoporosis in pregnancy and lactation and other risk factors [32]. Nutritional secondary hyperparathyroidism was associated with spontaneous fractures in cats and other animals [50,51]. Animal studies support using a multifactorial approach for osteotoxic nutritional risk factors.

The second category is ATF in MBDs with predominantly chronic hypokinesia disorder. Hypokinesia is a common sequence of medical disorders causing ATF including young people with neurologic disorders (spina bifida and cerebral palsy) [5,39], adults with neurologic disorders (hemiplegia, quadriplegia, paraplegia, dementia, Parkinson's disease) [18]; and arthropathies (e.g., rheumatoid arthritis) [14,52,53].

The third category is ATF in MBDs with predominantly osteotoxic hormonal or drug-induced disorders. Osteotoxic compounds causing ATF include L-thyroxine, antiepileptics, antidepressants, antipsychotics, proton pump inhibitors, loop diuretics, and warfarin [54-56]. Also, prolonged bisphosphonate usage causes the “atypical fracture of femur” (a challenging fragility fracture with ATF similarities). However, it was found to occur not only in femur but also not restricted to bisphosphonates. One example is a case with fracture cascade (tibia and femur) related to bisphosphonates, denosumab, and teriparatide [57]. This case had high OB from multiple risk factors, such as history of juvenile rheumatoid arthritis, osteotoxic glucocorticoid, low body mass index, hypokinesia after first fracture, and poor fracture healing. Bone biopsy showed normal bone volume, normal mineralization, and no osteomalacia. One study commented that “the Food and Drug Administration found no clear benefit of long-term use (>five years) of bisphosphonates in prevention of typical osteoporotic fractures” [58]. They should be managed wisely. Other hormonal causes of ATF are tumor-induced osteomalacia (FGF-23) and hyperparathyroidism [59,60]. ATF fractures with fracture cascades related to hyperparathyroidism (as in Case 3) were also reported in France and Brazil [61,62].

The fourth category is ATF in MBDs with multi-morbid frail (by clinical frailty scale) people [5,11]. These are the most common ATF cases in nursing homes. Cases have variable combinations of OB with no predominant risk factor. Reviewing the literature reveals that ATF is commonly a multifactorial disorder [11]. Perhaps ATF is better to be labeled as a “multifactorial MBD” or “multifactorial osteodystrophy.” Frailty definition or identification criteria might need revision based on the theory of the bone muscle unit and the ATF phenomenon [63].

Medicolegal challenges

ATF can cause serious medicolegal challenges suggestive of EAN by the caregivers or the healthcare providers and institutes. These fractures are occasionally described as unexplained, or suspected to be abusive, but prove to be non-abusive due to MBDs. Formal investigations found all five cases occurred during usual routine care (plus absence of EAN clinical and social features). This highlights the need for health education about ATF risk factors. American researchers emphasized that prior recognition that ATF is a not uncommon complication of prolonged bed rest, may lessen the confusion that often surrounds the response to such event [5]. Presentations challenges are as follows: ATF is highly correlated with a specified group of multi-morbid patients (even in the absence of osteoporosis due to its effect on bone mass quantity and quality) with hypokinesia, diabetes mellitus (DM), and contractures [64-67]. This group is described as a specialized group requiring customized care in order to encourage their remaining functions, which determine the quality of their residual life [68]. ATF is not an uncommon complication in people with dementia. Australian researchers stated that “dementia appears to be a marker of frailty and osteoporotic fracture risk” [69]. Chronic kidney disease was present in 3 of 5 cases of the present study, suggesting renal osteodystrophy contribution.

Case presentations varied, with one or more typical or atypical presentations (such as other geriatric syndromes). Atypical presentations are a challenge for diagnosis and may contribute to the unexplained fracture description. Communication difficulties are a challenge in ATF presentations (irritability or behavioral manifestations) in people with dementia, paraplegic children, and cerebral palsy cases [70,71]. Not considering atypical presentations causes dilemmas and over-investigations. Sudden give-way while standing was present in Case 4 of this study and 20 cases (2.5%) in a cross-sectional hip fracture study [20]. The orthopedics’ related acute phase response (APR) phenomenon was previously discussed but not correlated with ATF [72]. It includes a wide array of symptoms (such as fever, fatigue, anorexia, myalgia, and arthralgia). Bone fracture APR phenomenon was documented in ATF case reports and case series including paraplegic children ATF [18,61,70,73,74]. In murine femoral fracture, APR magnitude was correlated with the injury magnitude [75].

Case 2 had fracture-associated aseptic unexplained fever, which can be part of APR. In a Japanese case of unexplained fever and body pains with fracture cascades, fever was related to occult spontaneous fractures, same in pediatrics fractures were related to aseptic fever [76,77]. Osteogenic aseptic/non-malignant pyrexia seems to be a not uncommon phenomenon occurring in (A) fractures, (B) non-infectious postoperative orthopedics fever (a well-reviewed phenomenon [78]), and (C) intravenous zoledronic pyrexia [79,80].

Other atypical presentations are as follows: skin contusions or bruises are typical presentations in ATF cases [81-84]. However, it may cause a presentation challenge due to unexplained history (as in Case 2). ATF erythema and other APR findings were mistaken for osteomyelitis and in another case were misdiagnosed as deep venous thrombosis [71]. Painless long-bone ATF is another atypical feature (Cases 1 and 4 reported in spinal cord injury ATF patients) [70,71]. This indicates that ATF can occur painlessly even in ambulant people with limited mobility and perhaps resembling silent myocardial infarctions (in DM and older adults) or Charcot’s neuroarthropathy (in DM and polyneuropathy cases).

Lab and radiology challenges

Fragility fractures are investigated by a common battery (e.g., bone-specific laboratory; and radiology as bone densitometry). This helps in (1) excluding localized bone disorders, such as malignancy or cysts, and (2) identifying contributing morbidities. Occasionally, investigations were found to be normal [14]. It is difficult to attribute the expected or detected lab changes to a single disorder in multi-morbid older adults. The partial discrepancy in lab results might be explained by multi-morbidities interaction (e.g., osteoporosis, old occult fractures, malnutrition, vitamin D deficiency, organ impairment, malabsorption syndromes, and polypharmacy). For example, alkaline phosphatase increases with menopause, African Americans, high body mass index, and antiepileptics use [85]. Also, protein deficiency causes alkaline phosphatase levels to be low instead of high [86]. Negative plain x-ray does not exclude all fractures. An association between spiral fracture morphology and ATF was documented [5] (e.g., while transferring or turning cases [83,87], spinal cord injury cases [88,89], and cerebral palsy cases) [90-92]. Other investigations might be needed in ATF diagnosis [7].

Daily activities and raising awareness of ATF

ATF occurred during daily activities at home or in hospital, e.g., while rising from a chair. For bedridden cases, ATF can happen while turning, transferring, bathing, or any daily activity, as in the first, second, and third cases. Caregivers and healthcare providers should be educated about the occurrence of ATF including proper safe transferring techniques [5,6].

Warning signs for high risk of fractures

Two cases had a “fracture cascade” (having two or more fractures occurring in the same patient) [14]. The second osteoporotic fracture tended to occur one year following the initial fracture [93,94]. Avoiding this requires identifying at-risk group(s), managing modifiable OB (e.g., nutrition and safe transfer), and raising awareness about ATF. Inspired by the high risk of falls warning signs, we designed a “high risk of fracture” warning sign for individuals with high risk of ATF for adoption to decrease ATF incidents, fracture cascades, or medicolegal challenges (Figure 1). After designing the warning sign, we found Chinese fracture warning signs (simply written instruction signs) in a Taiwanese article [95] and safety report [96].

ATF management and outcomes

Conservative Management and Soft Casting

Conservative ATF management is a treatment of choice [88]. A fracture cascade, due to heavy back slab, occurred in the third case (while turning in bed) and was also reported in another case [70]. Soft casting was suggested as another possible treatment of choice, it might prevent fracture cascades. Atypical soft casting was used in ATF of paraplegic children [70], soft bulky splints [97], and soft plastic brace for spinal cord injury ATF patients [98].

Sound Bone in a Sound Body “Ossa Sanus in Corpore Sano”

The outcome in cases 1 and 2 was good, achieving healing with immobilization. Case 5 had an uneventful hip hemiarthroplasty and returned for her baseline activity. Case 3 died from sepsis and Case 4 lost to follow-up. A French study found a two-month mortality following long bone insufficiency ATF to be 24% in nursing homes [18]. Otherwise, in the authors' knowledge, there are no estimates of the morbidity, mortality, or ATF cost. However, it might be higher than the other osteoporotic fractures due to multi-morbidity. As a multifactorial disorder with high OB, treatment should correct as many risk factors as possible. When indicated, pharmacological and non-pharmacological treatment for osteoporosis is provided. It is important to manage common modifiable risk factors, such as nutrition, hypokinesia, osteotoxic medications, and non-pharmacological treatment in fragility fracture management [99]. We should always target “treating the whole body, not the sole bone” referred to as “Ossa sanus in corpore sano” sound bone in a sound body [99].

The present study has several limitations as a small case series retrospective study based on medical records. It highlights points for future research as osteogenic aseptic fever and atypical presentations of atraumatic fractures.

## Conclusions

Atraumatic fracture (ATF) is a complex underreported syndrome with epidemiological variation and occasional serious medical legality. One explanation is the variation in its terminology. Pathogenesis needs more studies to understand the relationship between bone quality and quantity. We should regard ATF as a manifestation of underlying multi-morbidity. Usually, the patients have a high osteotoxic burden (OB) causing metabolic bone disease(s). Understanding ATF should be based on the Utah paradigm, bone quality, and multi-morbidity. Incomplete management of comorbidities may cause failure of care and fracture cascades. The present study analyzed the OB and examined the challenges in ATF care. The case series showed high OB in all cases. We discussed new four broad categories for ATF etiological classification. We have correlated past literature with new ATF atypical presentations, such as the aseptic unexplained fever (as part of the bone-related acute phase reaction). We implemented a "high-risk of fracture" warning sign to prevent or decrease ATF in high-risk groups. Conservative management by soft casting is a possible ATF treatment choice. These points need further study on large-scale studies. As with all geriatric syndromes, it is important to raise awareness about "managing the whole body, not only the fracture." Through detecting and managing all modifiable OB. With the population aging and increase in multi-morbidity, the ATF syndrome needs further studies.
